# Determinants of Appropriate Child Health and Nutrition Practices among Women in Rural Gambia

**DOI:** 10.3329/jhpn.v28i2.4887

**Published:** 2010-04

**Authors:** Martha Mwangome, Andrew Prentice, Emma Plugge, Chidi Nweneka

**Affiliations:** ^1^ Kenya Medical Research Institute–Wellcome Trust Collaborative Research Programme, Centre for Geographic Medicine Research, PO Box 230, Kilifi, Kenya; ^2^ Medical Research Council Laboratories, Keneba Field Station, PO Box 273, Banjul, The Gambia, West Africa; ^3^ Department of Public Health and Primary Health Care, University of Oxford, OX3 7LZ Headington, Oxford, UK

**Keywords:** Child health, Child nutrition, Child nutrition disorders, Gender, Health education, Interventions, Knowledge, attitudes, practices, The Gambia

## Abstract

Health education and awareness involves providing knowledge about causes of illness and choices to promote a change in individual behaviour and, thus, improves survival of individuals. Studies have, however, shown that improved knowledge and awareness is not always translated into appropriate actions. This study aimed at exploring the factors determining mothers’ choices of appropriate child health and nutrition practices in the Gambia. Eight focus-group discussions (FGDs) were held with 63 women whose children had been seen at the Keneba MRC Clinic within the 12 months preceding the study. The FGDs were analyzed using a thematic framework. Gender inequality, presence or absence of support networks, alternative explanatory models of malnutrition, and poverty were identified as the main factors that would determine the ability of a mother to practise what she knows about child health and nutrition. The findings highlight the need to consider the broader social, cultural and economic factors, including the value of involving men in childcare, when designing nutritional interventions.

## INTRODUCTION

Malnutrition remains a factor in 60% of 11 million deaths of children aged less than five years globally each year. It is the most important risk factor for the burden of disease in developing countries (1), causing long-term detrimental consequences, such as impaired cognitive development, growth impairment, and poor academic performance (2). Children most at risk are those aged less than five years living in developing countries (3).

Recently, there has been an increase in the prevalence of malnutrition in Africa, which means that the goal set to reduce the levels of undernutrition by 50% between 1990 and 2015 may not be met. The number of underweight children in Africa increased from 26 million in 1990 to 32 million in 2000 (4). Other studies have predicted that the contribution to the global prevalence of childhood undernutrition from Africa will increase from 24.0% in 1990 to 26.8% in 2015 (5, 6).

To date, various interventions for targeting malnutrition have been proposed and implemented in different parts of the world. The use of health education as a component of child health and nutrition programmes is a common practice and is based on the premise that health-education messages promote specific behavioural changes, which should yield benefit in child survival. Studies have shown that nutritional knowledge of a mother is positively associated with the nutritional status of her children (7). Studies have also shown that the use of health education as a component is rarely sufficient on its own (8) and that adequate knowledge is not always translated into appropriate actions (9). Understanding the factors that determine the translation of adequate child health and nutrition knowledge into appropriate action might help design more effective interventions against malnutrition.

This paper presents the findings of a pilot study among women in a rural community in The Gambia. Detailed descriptions of the participating villages have been published elsewhere (10–12). These villages experience a seasonal agricultural system that revolves around an annual rainy season from July to November and a dry season from November to May (13). Data on maternal pregnancies, birth anthropometric measures, and gestational ages have been collected since 1978 (11, 13). Records of morbidity and anthropometric data on all children, aged less than three years, from the three villages, presenting at the field station's clinics, are also available in a database.

The Keneba MRC Nutrition Supplementation Centre, established approximately 20 years ago, is an example of both community-based management and nutrition/health-education approaches for the rehabilitation of severely-malnourished children. The activities at the Keneba field station, including those of the supplementation centre, have directly or indirectly exposed the community to a substantial amount of child health and nutrition knowledge and awareness. Some of these activities include provision of clinical care and nutritional supplementation to severely-malnourished children, education of carers on child-nutrition practices, basic hygiene, and food-preparation methods. Other activities include various nutrition-related research and intervention programmes the station has supported since its founding (14–16). These interventions have been thought to contribute to the reduction of the risk of dying by as much as three times in infants aged 0–1 years and up to seven times in all children aged less than five years in rural Gambia (15, 16), although this improvement in mortality rate was not necessarily associated with improvements in the nutritional status of children (11).

The aim of this pilot study was to explore the factors determining mothers’ choices of appropriate child health and nutrition practices in this community. The term ‘appropriate’ practice is used in the title and text to mean practices that enable the child to grow and develop normally, additionally, those that are not detrimental to the well-being of the child.

## MATERIALS AND METHODS

### Study site

The study was undertaken among mothers in three villages near the Keneba field station of the Medical Research Council (MRC) in the Gambia. The three villages—Keneba, Manduar, and Kantong Kunda—are commonly referred to as the core villages because they were the original sites of research activities of the Keneba field station.

### Participants

The study involved mothers of children, aged less than three years, residing in any one of the three core villages and had been seen at the MRC clinic in the 12 months before the commencement of the study (March 2005–February 2006). The MRC surveillance database was used for identifying these children and their mothers.

Purposive sampling was used for identifying and selecting a homogenous group of participants for the FGDs. It was assumed that this group of women would give up-to-date information of the situation in question since they would be going through the process of making feeding choices at the time of the study. They were also preferred because the available dataset at the MRC could easily be used for identifying them.

Verbal informed consent was obtained individually for participation. The MRC Gambia Scientific Coordinating Committee and the Gambian Government/MRC Joint Ethics Committee approved the study.

### Focus-group discussions

An FGD guide was developed in line with the main research question: “why is it that the mothers in this community cannot practise what they know about child health and nutrition?” The discussion was guided by the existing literature on the subject “Does adequate knowledge lead to appropriate action?” and through conducting informal discussions with the local health workers around the Keneba field station. It contained unstructured open-ended questions with non-directive prompts used for generating and directing the discussion.

Informed consent was obtained from the mothers for tape-recording the discussions. Two trained, experienced nurses fluent in Mandinka—one as the facilitator and the other as a note-taker—conducted all discussions in this local language. One of the authors (MM) observed all the sessions.

### Analysis of data on focus-group discussions

The transcripts from the eight FGDs were prepared by the facilitators, translated into English and entered separately as Microsoft word documents. Thematic framework approach (17) was used for analyzing the transcripts. This involved reading through the transcripts and identifying emerging themes which were grouped into main themes (concepts) and sub-themes as other new themes emerged.

## RESULTS

### Study participants

Of the 70 mothers invited for the FGDs, 63 participated in the discussions. Each focus group involved 7–8 mothers. The mean age and parity of the women was 32 years and five children respectively. The level of education among the participants was generally low. Eleven of the mothers had formal education while 34 had attended Arabic school at some point in their life. The rest had non-formal education. Further information on individual social economic status was not sought; however, detailed description, including general social economic status of the inhabitants of the three research villages, has been published elsewhere (10–12).

### Themes emerging from focus-group discussion

The study explored the factors determining mothers’ choices to appropriate child health and nutrition practices in this rural community. Several factors emerging as themes are described below.

#### Gender role inequality

The role of men and women in this society as described by the participants indicates that women are the sole caretakers of their children with little or no assistance from their male counterparts. They (women) engage in laborious work under harsh conditions sometimes at the expense of the child's welfare.

Women are expected to do both farm work and household chores, thus, infringing on the time allocated for the health and nutritional needs of the child as one mother reported:

They (our husbands) should be helping us but unfortunately they are not doing it. What can one do when a man says no!

Women also reported that men receive the largest, best, and first share of the meals and that the women only eat after the men and children are satisfied. This practice was attributed to their respect for their husbands:

The men are our husbands, and we respect them and so give them a larger share. They are the leaders, and so, we give them the best parts.

This cultural practice may have severe consequences on the health and nutrition of pregnant mothers and consequently the child they bear.

The women also reported that decision-making on issues of marriage, child-bearing, and child-spacing were out of their domain, although these affect them. Such decisions in many households in this community are either strongly influenced or are made solely by men. They further reported incidences where men would have the last word on issues without necessarily consulting or considering the women's opinions. Thus, the knowledge acquired by women during clinical visits would not necessarily lead to the intended response if it were in conflict with the views of men.

#### Poverty

The impact of poverty on malnutrition in these communities was highlighted in various ways: (a) poverty leading to lack of food, or lack of variety of foods; (b) poverty aggravating the inequalities and desperation already experienced by women in the community; (c) lack of alternative means of livelihood making the women vulnerable to harsh environmental conditions, which further aggravate their food situation. Such conditions may include insects and wild animals attacking and destroying farm crops. One women stated:

It is beyond us. Each of us would want to stay at home with our children but we have to survive. The only way for us to survive without begging is to farm. We do not like it that way but we have no choice.

#### Alternative explanatory models of malnutrition

The study women identified alternative explanations to causes of nutrition-related ailments among children in this community. These alternative explanations are closely related to cultural beliefs and perceptions about the origin and treatment of malnutrition. The theory of the ‘devil child’ is one such explanation that blames malnutrition for an evil spirit. The characteristics, causes, and treatment of the devil child were described in detail by the women during the discussions. It was noted that the characteristics of the devil child included:

A child usually born with a big head and a small wrinkled body resembling features of a small-for-gestational age baby or a low-birthweight baby.

The women described the causes and treatment of this syndrome. They described how, if the disease is perceived to be caused by the devil, the treatment would be to test the infant for its humane nature and prove that the child is not a devil:

… mothers will take such a child to the bush, place the child somewhere, and then leave for a few hours. If the mother returns and does not find the child, she will know that the child was really the devil but if the mother finds the child where she left him, she will know that it is a human being, she will take the child home, and continue treating it.

Other than the devil child, the women discussed other conditions with physical resemblance to those of a malnourished child. Such conditions are locally known by the names—*montoo, tiyoo,* and *fonoo. Montoo* was described as a disease causing a child to have high fever, a big pot-shaped abdomen, and brown hair while *tiyoo* was a disease associated with the rainy season while *fonoo* was associated with bad wind, blown by evil spirit. Treatment for these ailments was reported to occur at the local herbalist and, according to the mothers, would depend on the perceived cause of the disease.

If a child has *tiyoo or montoo*, you go to the herbalist for treatment, for *fonoo,*—a dry bone from a dead crocodile is soaked in water, and then the child is washed with that water.

#### Role of support networks

Social support in this context is used for referring to the degree to which the physical and emotional needs of women are satisfied through their interaction with organizations and societies (formal) and relatives, friends, and others (informal).

The women identified their husbands, older siblings to the index child, and in-laws (grandmothers to the child) as part of child health and nutrition-support networks. They identified circumstances where both presence and absence of the support networks in child health and nutrition might be experienced. For example, it was explained that:

When mothers go to the field, the child is left with other children, or at times, we will leave them at home under the care of an elderly person.

The mothers felt supported in childcare by the presence and the working system of the MRC clinic in Keneba village:

The nurses have shown us how weights are plotted on the charts. If the child is loosing weight, they will advise you on diet.

Economic support was reported in areas of farming and farm-products through friends and relatives and also in the activities of non-governmental organizations through providing farm-equipment:

… There was a time that the wife of an MRC's doctor sent a group to us that brought some farm-equipment ….

In this regard, the presence or absence of the support network has direct or indirect influences in ensuring proper care for the child.

### Public-health implications of study results

We explored the factors that determine mothers’ choices of appropriate child health and nutrition practices. Our findings suggest that the key determinants include gender relations within the family, presence and types of support networks available to the mother, alternative explanatory models of malnutrition within a particular community, and the availability of food in the communities. These findings have public-health implications.

Gender relations with respect to decisions on child health and nutrition as found in this study are consistent with reports from other parts of the world (18). First, in a setting where a husband makes the key decisions, claims top priority in household resource allocation, including food, and is in total control of household finances, the mother has limited choices when deciding on appropriate child-health and nutrition practices (18).

In such settings, giving mothers adequate knowledge on proper childcare practices has a little impact on consequent actions without the involvement of the partner.

Second, men mediate on access of women to economic resources, which has implications on the nutritional status of women, especially during pregnancy and nursing periods. This means that the health of both child and mother depends heavily on the decisions of the dominant male figure in their life (19).

Third, men were reported to dictate decisions on child-spacing and family size, which may ultimately affect the health and nutrition of children (20). The rigidity and ignorance of men on issues of family planning hinders the fertility decline in four ways: (a) by dominating in reproductive decisions for which they are ignorant; (b) by refusing to provide economic resources required to access contraception; (c) by displaying inflexibility towards family-planning initiatives as reported in other studies (19, 21); and (d) men were found to dictate on the timing and choice of the marriage partner for women. This makes it impossible for the woman to negotiate her own child-bearing practices and discuss other child-upbringing choices with the man.

Poverty is a complex multi-dimensional phenomenon characterized by deprivation, exclusion, and vulnerability. The results showed a number of aspects of poverty in the context of their children's health and nutrition practices. First, poverty as lack of food demonstrates the direct link to under-nutrition in most communities around the world. In fact, malnutrition is a legitimate indicator of poverty in society (22). Second, poverty as powerlessness is aggravated by the role of gender bias within the community. The results showed that women lacked a sense of social power and financial control over their households and were, therefore, unable to negotiate for proper food choices for their children (1). Lastly, poverty as vulnerability is seen in the susceptibility of the community's food situation to environmental conditions. This community's survival is completely dependent on production of crops, which, in turn, is reliant on environmental conditions, making them highly vulnerable to climate change and other adverse environmental circumstances. This finding is consistent with those of other reports (23).

Although the traditional beliefs and practices as discussed in the present study may not be in current practice among members of this community, of importance is that the participants felt the need to point them out as factors that may determine their choices of proper child-nutrition practice. This shows that the factors may still have an influence on the way of thinking and, thus, on the decision-making process among the community members. The traditional beliefs and practices could affect child health and nutrition in different ways: (a) they could interrupt and or complicate the disease status of children to the point of death or disability; (b) they could deplete or limit the available family resources, thereby limiting the capacity of families to pay for proper biomedical interventions; (c) they could breed social stigma, in this case ‘the devil child’ which could lead to negligence and improper treatment of an otherwise malnourished child.

We also identified the cultural beliefs and practices that encourage good hygiene and childcare practices, such as protecting children from extreme wetness. *“Tiyoo is a condition that occurs during the rainy season when a child is put to sit on the floor or wet ground.”* The need to identify and address the cultural rationales that underlie negative practices and to reinforce and protect the beliefs that support positive practices has been highlighted in other studies (24).

Poor social support networks as reported in this study and the huge domestic and agricultural demand on the mother expose the child to maternal deprivation, which, in turn, impacts negatively on the child's health. As part of the support networks in rural Africa, elderly women, especially grandmothers and even siblings, are often called upon to baby-sit when the mother needs to be away. Grandmothers are trusted to be skilled in child upbringing, irrespective of the existence of the generation gaps (21). They are preferred for their perceived child-upbringing skills and know-how. Studies have shown that having a living maternal grandmother has a significant effect on the survival of the child (25, 26). In rural communities, grandmothers command respect from their sons (women's husbands). This changes the dynamics of the decision-making process in a household. This is true in scenarios where the man (father to the index child) is mostly away. In such settings, the mother might have adequate knowledge to inform proper action but her actions are highly influenced by the grandmother's opinion (22, 25).

### Limitations

Qualitative research was undoubtedly the most appropriate method of gaining an understanding of the child health and nutrition practices of mothers. However, the investigators were not fluent in the local language by whom most data were collected and, therefore, required the use of trained local staff for the translation of the transcripts. The complexity of translating non-English focus-group data may have introduced unidentified bias (27).

### Conclusions

Malnutrition is an important global public-health problem, and it is important to understand the factors that determine the translation of child health and nutrition knowledge into appropriate actions to develop more effective interventions against malnutrition. This study has shown the potential positive impact of considering gender equity and poverty-reduction initiatives as interventions to improve child health and nutrition. Our findings also concur with those of earlier reports that malnutrition is a product of complex interplay between many different factors, some of which are social factors such that any successful interventions will benefit from considering the broad cultural and socioeconomic context in which the intervention is to be delivered.

## ACKNOWLEDGEMENTS

The authors acknowledge the support of Bakare Kante, Fatumata Sanyang, Fatumata Sidibeh, Sira Bah, all the staff at MRC Keneba and the community mothers, children, and families who willingly participated in the study.
